# Postoperative diaphragmatic hernia following endoscopic thoracic sympathectomy for primary palmar hyperhidrosis: A case report

**DOI:** 10.3389/fsurg.2022.1059604

**Published:** 2023-01-06

**Authors:** Ling Wang, Xike Wu, Yuepu Tang, Zheyuan Fan

**Affiliations:** ^1^ Department of Emergency Medicine, The First Affiliated Hospital of Dalian Medical University, Dalian, China; ^2^ Department of Cardiothoracic Surgery, The Affiliated Jinyang Hospital of Guizhou Medical University, Guiyang, China

**Keywords:** primary palmar hyperhidrosis, sympathectomy, diaphragmatic hernia, case report, video-assisted thoracoscopic sympathectomy

## Abstract

Postoperative diaphragmatic hernia (DH) following endoscopic thoracic sympathectomy for primary palmar hyperhidrosis is extremely rare. We present a 21-year-old female patient who developed a left DH with herniation of the stomach and gastric perforation on the first postoperative day after undergoing bilateral video-assisted thoracoscopic sympathectomy R4 ablation. She complained of severe dyspnea and chest pain, and an emergency chest x-ray and computed tomography revealed left pleural effusion, collapsed lung, and left DH, which allowed the stomach to herniate into the chest. Emergency thoracoscopic surgery was performed. We repaired the diaphragmatic defect intraoperatively and replaced the stomach with the peritoneal cavity from the thoracic field. The patient was discharged without complications. She did not present with recurrent symptoms at the 3-month follow-up. Postoperative DH should be considered when patients complain of gastrointestinal or respiratory symptoms after sympathectomy, although it is very rare.

## Introduction

Primary palmar hyperhidrosis (PPH) is defined as a pathologic condition of excessive sweating over 6 months in duration that impairs daily activities without occurring secondary to other specific diseases or medications (1). Currently, endoscopic thoracic sympathectomy (ETS) is an effective therapeutic method for the treatment of PPH (2), but there are also some common complications, including Horner's syndrome, pneumothorax, and hemorrhage, which have been reported worldwide (3). A few cases of diaphragmatic hernia (DH) or tension gastrothorax as a complication of thoracic and abdominal surgery occur (4, 5). DH after ETS has not been reported before, which leads to misdiagnosis or late diagnosis, resulting in high morbidity and mortality rates.

Here we report an extremely rare case of left DH with herniation of the gastric fundus and body following bilateral video-assisted thoracoscopic sympathectomy (VATS) R4 ablation for PPH, and the patient's primary clinical manifestation was respiratory distress. We successfully repaired the DH and the stomach defect by thoracoscopy with mesh placement.

## Case presentation

A 21-year-old female patient without any specific medical history presented at our hospital with excessive palmar sweating for 10 years. She had previously received conservative treatments from a local hospital, including topical antiperspirants containing aluminum chloride hexahydrate; however, the symptoms were not relieved. We evaluated the severity of the disease based on the Hyperhidrosis Disease Severity Scale (HDSS) (6), and the preoperative diagnosis was severe PPH. A bilateral video-assisted thoracoscopic sympathectomy R4 ablation was recommended for the patient. The patient was administered general anesthesia and double-lumen endotracheal intubation. The patient was positioned supine in a semi-sitting position with the arms abducted 90°. A 1-cm access port was inserted in the midaxillary lines over the third intercostal space with a 30°, 10-mm video camera placed in the anterior and a 5-mm endoscopic hook placed in the posterior. No CO_2_ insufflation was used for exposure. The chest was visualized, and the sympathetic chain was identified. Sympathotomy was performed with an electrocautery section of the sympathetic chain over the head of the rib, extending the burn along the rib for a length of 2–3 cm to cauterize potential bypassing branches of the chain (nerve of Kuntz). The level of sympathotomy (R4) was performed according to Chinese expert consensus (7). After lung expansion, no chest tube was routinely left in the thoracic cavity. The procedure was performed successfully, and sweating from the hands stopped immediately.

**Figure 1:**
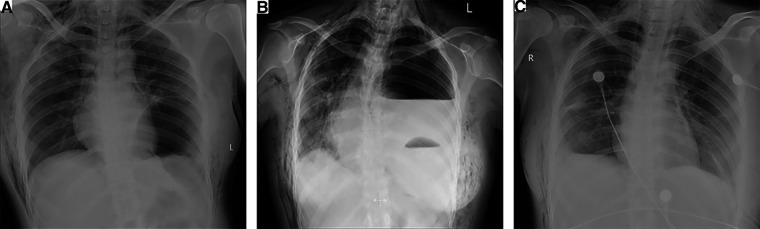
Chest radiographs. (**A**) Initial chest radiograph revealing an elevated left hemidiaphragm and pneumothorax in the left lung. (**B**) Chest radiograph showed left pleural effusion and a mediastinal shift toward the right with an elevated left hemidiaphragm. (**C**) Postoperative chest radiograph revealed normal positions of the stomach bubble and diaphragmatic contour.

**Figure 2:**
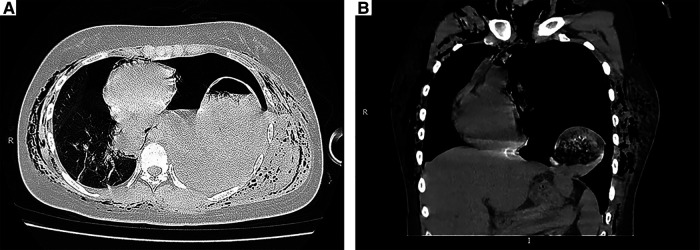
CT scans. (**A**) Preoperative chest CT image showed left pleural effusion, collapsed lung and intragastric gas with an air-fluid level in the left thoracic cavity. (**B**) Coronal CT image demonstrating that the stomach had migrated into the thorax.

On the day of surgery, she began to complain of epigastric discomfort and vomiting, and fasting therapy was given. Chest radiography revealed a pneumothorax in the left lung with an elevated left hemidiaphragm (Figure 1A). The following day, the patient complained of severe dyspnea and chest pain. On chest auscultation, low breath sounds were heard on the left side, and the systemic examination was normal. Chest x-ray revealed a left pleural effusion and a mediastinal shift toward the right with an elevated left hemidiaphragm (Figure 1B). Thoracocentesis was performed, both for diagnostic testing and drainage of the pleural fluid; and 60 ml of brown fluid, clinically suggestive of gastric juices, was drained by a needle. Emergency chest computed tomography (CT) demonstrated left pleural effusion, collapsed lung, and left DH, which allowed the stomach to herniate into the chest (Figures 2A,B). We diagnosed left DH incarceration and performed an emergency thoracoscopic repair of the hernia.

**Figure 3:**
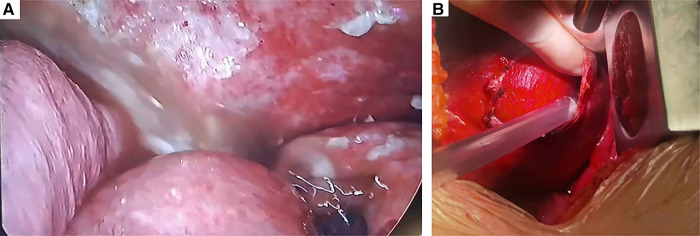
Intraoperative findings of the second operation. (**A**) Contamination of the thoracic cavity with stomach contents. (**B**) Intraoperatively, a 6-cm-diameter diaphragmatic defect was found.

Emergency surgery was performed, and the patient was administered general anesthesia with double-lumen endotracheal intubation and placed in the right hemilateral position for surgery. The operation was performed thoracoscopically and conducted through the eighth intercostal space to expose the pleural cavity. Intraoperatively, a contaminated thoracic cavity with stomach contents was observed, without pleural adhesions (Figure 3A). There was an approximately 6-cm diaphragmatic defect in the left posteromedial diaphragm, and the stomach had a 1-cm rupture and was herniated into the thorax through the defect (Figure 3B). There were no obvious ischemic findings. Abundant irrigation and direct repair of the damaged stomach and replacement of it into the peritoneal cavity from the thoracic field were performed by the general surgery team. We repaired the diaphragmatic defect with nonabsorbable sutures and reinforced it with polypropylene hernia repair mesh all around the defect. The operation was completed after the placement of drains in the thoracic cavities. The postoperative chest x-rays were normal, and the patient was discharged in good condition 10 days after the second operation without complications (Figure 1C). The patient did not receive any other medical treatment, and there were no other concomitant medical conditions requiring attention. The 3-month follow-up confirmed the absence of symptoms. At the time of this writing, her sweating had stopped for approximately 8 months, and there were no adverse effects during that period. Written informed consent was obtained from the patient.

## Discussion

Thoracoscopic bilateral dorsal sympathectomy is the standard therapeutic method for PPH. To our knowledge, DH is a rare complication of sympathectomy and leads to life-threatening cases of strangulation or perforation as well as cardiovascular and respiratory insufficiencies (8). In this case, the patient complained of severe dyspnea and chest pain after ETS and was diagnosed with left DH with herniation of the gastric fundus and body. We repaired the damaged stomach and replaced it in the peritoneal cavity with VATS alone. In this case, we believe that the thoracic approach is more advantageous for the following reasons: (1) the thoracotomy wound that was used for the endoscopic orifice in the endoscopic thoracic sympathectomy can be used again; and (2) the patient complained of severe dyspnea and chest pain on the second day after the first operation. Considering the complexity of intrathoracic surgery and potentially serious complications, we chose the thoracoscopic approach in this case; (3) the preoperative images showed no obvious free gas under the diaphragm and additional intra-abdominal organ injuries.

The majority of the patients with diaphragmatic defects have defects that remain small, and they only complain of gastrointestinal symptoms (such as vomiting, postprandial discomfort, nausea) and respiratory symptoms (such as chest pain, cough, dyspnea). Patients can be asymptomatic for a long time and be diagnosed with delayed iatrogenic DH after surgery (5). Patients with DH incarceration and rupture of hernia contents can be critically symptomatic immediately after surgery, such as in our case, complaining of dyspnea on the first postoperative day.

Considering the pathogenesis of DH in our case, it could be related to congenital weakness of the diaphragm. Moreover, prolonged anesthesia induction led to gastric pouch dilatation and continuous high airway pressure, which might amplify the transabdominal-pleural cavity pressure. The dilated gastric wall compressed the left diaphragm, and prolonged intense compression caused severe ischemia, which decreased the elasticity and strength of the diaphragm and eventually led to rupture of the left diaphragm and the formation of an incarcerated diaphragmatic hiatal hernia. During the surgery, the patient is intubated with positive pressure ventilation in the chest, and small perforations in the diaphragm remain collapsed and prevent the migration of abdominal structures. However, in the postoperative period, the respiration and the pressure gradient between the pleural cavity and abdomen consistently pull the small, defective diaphragm radially, gradually extending the small orifice over time until it allows abdominal organ herniation, especially on the left side, because of the cushioning effect of the liver protecting the right hemi diaphragm.

Several reports have shown that initially, the chest x-ray is normal or can mimic pleural effusion, pneumonia, or pneumothorax, which can lead to a misdiagnosis. CT is the imaging modality of choice; whenever we see a chest x-ray or CT suggesting obscured diaphragmatic shadow, irregularity of the diaphragmatic contour, pleural effusion, and mediastinal shift, we should suspect the possibility of DH (9).

There are several limitations in our approach to this case. First, on the day of the first surgery, the patient began to complain of nausea and vomiting, and we were not aware of the risk and clinical presentation of diaphragmatic hernia. Second, we should use emergent gastric decompression with a nasogastric tube that may control the situation and let us buy some time to save the patients with fluids and acid–base balance adjustment. Third, the deficiency of thoracentesis is the lack of ultrasound guidance, which is dangerous in such situations. Fourth, a total follow-up period of 3 months by a surgeon may be too short to evaluate the prognosis of the patient.

In conclusion, it is important that thoracic surgeons inspect the integrity of the diaphragm at the end of surgery and consider the possibility of this rare complication in patients presenting with gastrointestinal or respiratory symptoms, especially after a left-sided thoracic procedure. After confirmation of DH, a feasible and reliable thoracoabdominal approach could be immediately used for treatment, including reduction of herniated organs and progression to more serious complications (10).

## Data Availability

The original contributions presented in the study are included in the article/Supplementary Material, further inquiries can be directed to the corresponding author.
